# INTEGRATING CERTIFIED NURSING ASSISTANTS INTO INTERDISCIPLINARY CARE TEAM AND RESIDENT CARE PLANNING

**DOI:** 10.1093/geroni/igae098.4266

**Published:** 2024-12-31

**Authors:** Aasha Raval, Mia Ermolenko, Andreina Jimenez, Anisha Balaji, Shivani Shenoy, Marissa Bergh, Jasmine Travers

**Affiliations:** New York University, New York, New York, United States; SUNY Downstate College of Medicine, Brooklyn, New York, United States; New York University, New York, New York, United States; Albert Einstein College of Medicine, New York, New York, United States; New York University, New York, New York, United States; New York University, New York, New York, United States; New York University, New York, New York, United States

## Abstract

Integrating certified nursing assistants (CNAs) into interdisciplinary care team (IDT) and resident care planning is essential for delivering person-centered care in nursing homes. In 2016, the Centers for Medicare and Medicaid Services mandated this integration, but the lack of clear guidelines made implementation challenging. As a result, a specific road map is needed for nursing homes to optimize how best to integrate CNAs into IDT and resident care planning. This qualitative study, guided by Donabedian’s Quality of Care Model, explored the barriers and facilitators in integrating CNAs into IDT and resident care planning. Seventeen participants including CNAs, directors of nursing, social workers, nursing home surveyors, ombudsmen, and physicians were recruited from across the United States using the State Survey Agency Directory, LinkedIn, and a referral program initiated by our research team. One-on-one, 30-minute virtual Zoom interviews were conducted between June-2022 and June-2023. Interview data were analyzed using directed content and thematic analyses. Five key themes emerged from the data and included: recognition of challenges for CNA Integration, nursing home leadership and structure, integration of CNAs, care coordination and communication, and care integrity. The findings led to the development of two INTEGRATE guides in August 2024: one for nursing home executive leadership which offers strategies for effectively integrating CNAs into IDT and resident care planning, and another for CNAs, which provides practical guidance on contributing to IDT meetings and care planning processes. The next steps of this work involve testing the efficacy and acceptability of the INTEGRATE guides in nursing homes.

